# Negative regulation of human IL-33 in endothelium during allergic airway inflammation

**DOI:** 10.1172/jci.insight.190418

**Published:** 2026-05-08

**Authors:** Maile K. Hollinger, Chanie L. Howard, Donna C. Decker, Kelly M. Blaine, Ivy Aneas, Emily M. Grayson, Tania E. Velez, Fernando A. Oliveira, Riley T. Hannan, Daniel F. Camacho, Philip A. Verhoef, Cara L. Hrusch, Rebecca S. Griffes, Jeffrey M. Sturek, Marcelo A. Nobrega, Nathan Schoettler, Anne I. Sperling

**Affiliations:** 1 Committee on Immunology and; 2 Department of Medicine, Section of Pulmonary and Critical Care Medicine, University of Chicago, Chicago, Illinois, USA.; 3 Division of Pulmonary and Critical Care Medicine, School of Medicine, University of Virginia, Charlottesville, Virginia, USA.; 4 Department of Human Genetics, University of Chicago, Chicago, Illinois, USA.; 5 Department of Microbiology, Immunology, and Cancer Biology, University of Virginia, Charlottesville, Virginia, USA.; 6 Cellular and Molecular Neurobiology Laboratory (LaNeC), Center for Mathematics, Computing and Cognition (CMCC), Federal University of ABC – UFABC, São Bernardo do Campo, São Paulo, Brazil.; 7 Kaiser Permanente Moanalua Medical Center, Honolulu, Hawaii, USA.

**Keywords:** Immunology, Vascular biology, Allergy, Asthma, Cytokines

## Abstract

Lung IL-33 is involved in pathogen defense, barrier homeostasis, and development of allergic responses. We previously identified a 5 kb noncoding region within a GWAS-defined segment that regulates expression of human *IL33* (h*IL33*) but is absent in the murine locus. To understand how this region affects IL-33 expression in vivo, we engineered 2 BAC-transgenic strains in which 166 kb of the human genome upstream of the *hIL33* locus, along with a fluorescent reporter, was inserted into the murine genome, both with and without the 5 kb region. Comparison to a murine *Il33* (m*Il33*) reporter strain revealed species-specific tropism; h*IL33* reporter was mostly expressed in the endothelium, while m*Il33* reporter was expressed in type 2 alveolar epithelium. h*IL33* reporter expression in tracheal basal epithelium, submucosal glands, and lung microvasculature required the 5 kb region. Surprisingly, allergen and exogenous IL-33 downregulated h*IL33* reporter in lung endothelium only when the 5 kb region was present. Similar IL-33–dependent downregulation of *IL33* transcripts was observed in human endothelial cell lines, validating that our h*IL33* reporter strain recapitulated human endothelial biology. Together, these data reveal the importance of the asthma-associated human 5 kb region in regulating human *IL33* expression in a cell type– and context-dependent manner.

## Abstract 2

Employing a genomic human *IL33*-reporter strain, we identify a negative feedback loop for endothelial *IL33* expression in response to either allergen or exogenous IL-33.

## Introduction

Asthma is a heterogeneous respiratory disease that is affected by a multitude of genetic and environmental factors (1–3). While healthy individuals do not mount immune responses to inhaled allergens, others develop an asthmatic response characterized by airway hyperreactivity and eosinophilic inflammation. Since 2007, numerous genome-wide association studies (GWASs) in ancestrally diverse cohorts have been used to identify asthma susceptibility alleles and to elucidate the complex interplay between genetic variability and environmental exposure (4). The nuclear cytokine IL-33 is released from barrier tissues upon stress or injury, such as degradation by allergen-associated proteases (5). Unsurprisingly, single-nucleotide polymorphisms (SNPs) near the genes encoding the alarmin *IL33* and its cognate receptor ST2 (*IL1RL1*) have been strongly associated with asthma in GWAS. Lungs from individuals with asthma express higher levels of *IL33* transcript than healthy controls, further suggesting the importance of IL-33 in clinical asthma (6).

Mechanistic studies of IL-33 in mouse models have contributed greatly to our understanding of its role in initiating and exacerbating allergic asthma. For example, IL-33 activates ILC2s upstream of airway hyperreactivity (7), mediates crosstalk between mast cells and airway smooth muscle cells (8), and drives recruitment of monocytes into the lung during allergic inflammation (9). However, translating biological insights from murine models into clinical therapies has been difficult. Specifically, a monoclonal antibody developed to block IL-33 demonstrated great promise in ameliorating airway hyperreactivity in an HDM-induced murine model of allergic asthma (10). However, in phase 2 clinical trials, IL-33 blockade was less effective at controlling asthma than the IL-4/IL-13 blockading antibody dupilumab (11). Thus, differences between the biology of IL-33 in mice and humans may preclude direct translation into therapeutic benefit, and studies of these differences may uncover better strategies for modulating IL-33 in the clinic.

One of the notable differences between human and mouse IL-33 is lack of genome sequence conservation in noncoding regions. We previously showed that a key 5 kb region upstream of the human *IL33* gene, containing asthma GWAS SNPs, is absent from the murine *Il33* locus. To understand how this 5 kb regulatory region affects *IL33* expression in vivo, we generated a bacterial artificial chromosome (BAC) containing the entire human *IL33* locus, including upstream and downstream regulatory regions of human *IL33,* with insertion of a Crimson reporter protein and stop codon into the second exon of *IL33*, thereby preventing expression of full-length human *IL33*. Mice containing this BAC demonstrate *IL33*-reporter staining in lymph node (LN) high endothelial venules (HEVs), consistent with the expression of *IL33* transcripts in human LNs (12). Unlike human IL-33, murine IL-33 is constitutively expressed in epithelial cells from barrier tissues such as the vagina, skin, lung, stomach and salivary glands, but it is absent from HEVs (13). Given that *Il33* expression patterns in murine models do not recapitulate human *IL33* expression patterns, it is essential to understand how differential regulation of IL-33 in mice and humans can account for discrepancies between murine experimental models and IL-33 function in human clinical settings.

Here, we find that, unlike murine *Il33* reporter, which is expressed largely by alveolar epithelial type II cells (ATII), human *IL33* reporter is constitutively expressed by lung endothelium. To our surprise, allergic sensitization with house dust mite (HDM) downregulated human *IL33* reporter in the endothelium, and this downregulation was dependent on the 5 kb regulatory region. In human endothelial cells, we also observed IL-33–dependent downregulation of *IL33* transcripts, underscoring that IL-33–dependent regulation of *IL33* reporter in our BAC model reflected endothelial biology in human cells. Together, these data reveal the ability of human IL-33 to control its own transcript levels in the endothelium during allergic airway disease and pinpoint a region in the human *IL33* locus that underlies this distinct response.

## Results

### Human and mouse IL33 reporters are expressed by distinct cell populations in the lungs.

**Figure 1:**
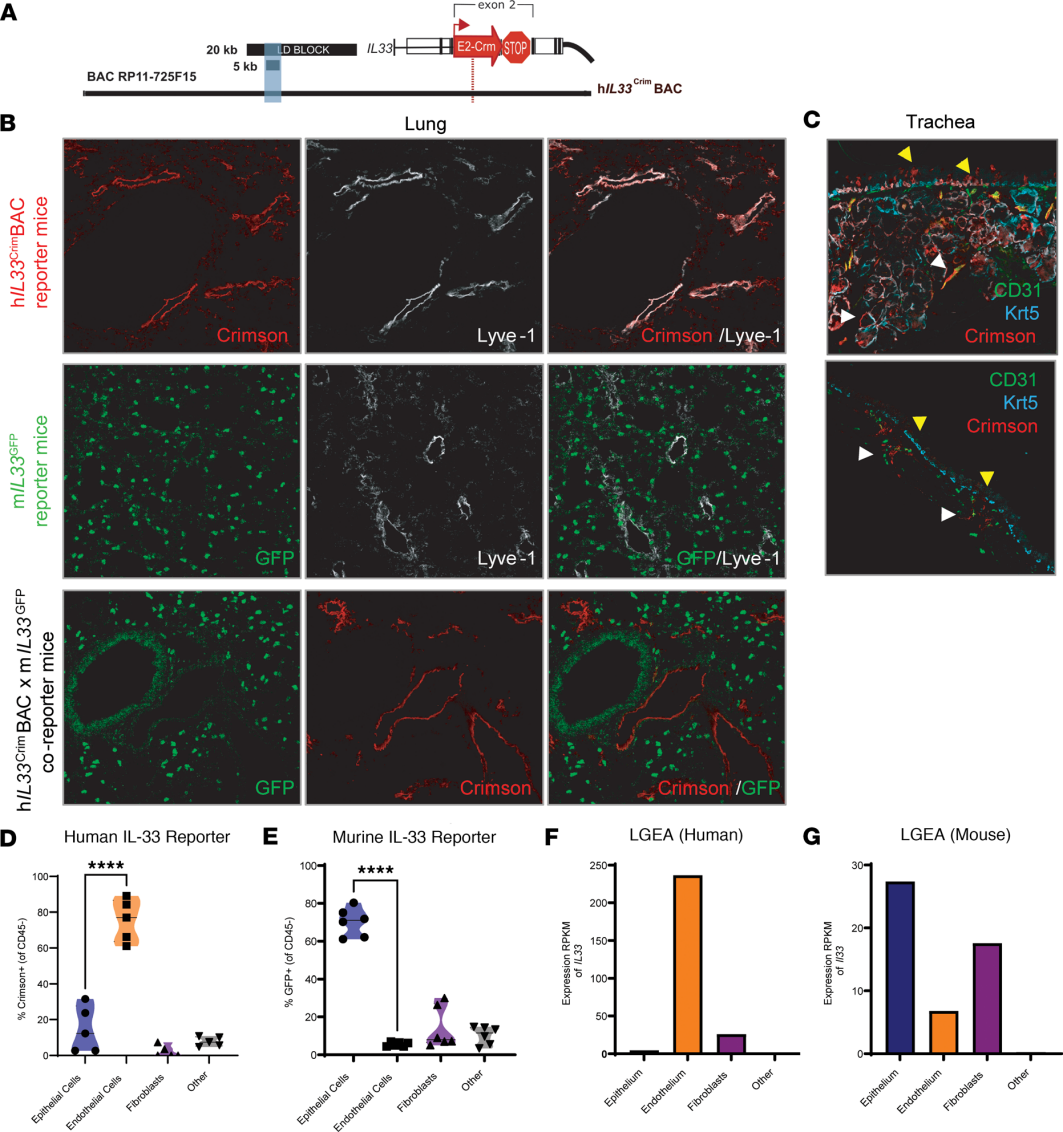
Human and mouse *IL33* reporters are expressed by distinct cell populations in the lungs. (**A**) Diagram of the BAC construct used to generate h*IL33*^Crim^BAC reporter mice. Black rectangles indicate LD blocks containing causal asthma-associated SNPs described previously (16). In the coding region of human *IL33*, we inserted an E2-Crimson coding sequence and stop codon to prevent expression of human *IL33* in BAC-containing mice. (**B**) Confocal microscopy (40×) of lung from 6- to 8-week-old naive h*IL33*^Crim^BAC reporter mice (top row), m*IL33*^GFP^ reporter mice (middle row), and h*IL33*^Crim^BAC x m*IL33*^GFP^ coreporter mice. Red indicates Crimson, green indicates GFP, and white indicates Lyve-1, an endothelial cell marker. (**C**) Confocal microscopy (40×) of tracheas from naive h*IL33*^Crim^BAC reporter mice (top) or m*IL33*^GFP^ reporter mice (bottom). Green indicates the endothelial cell marker CD31, cyan indicates the basal epithelial cell marker Krt5, and red indicates Crimson in h*IL33*^Crim^BAC or GFP in m*IL33*^GFP^ mice, respectively. Yellow triangles: Krt5^+^ tracheal epithelium; white triangles: submucosal glands (SMG). (**D**) Quantification of Crimson human IL-33 reporter fluorescence in lung CD45^–^ cells as gated in Supplemental Figure 2. (**E**) Quantification of GFP (murine IL-33 reporter) fluorescence in lung CD45^–^ cells. (**F**) Expression of *Il33* mRNA in sorted CD45^–^ cells from 20-month-old human lung donor from the LGEA. (**G**) Expression of *Il33* mRNA in sorted CD45^–^ cells from mice postnatal day 28 from the LGEA. Quantified data are represented as mean ± first and third quartile. Data in **D** and **E** depict 2 of at least 3 independent experiments, with *n* ≥ 2 mice per group. ****P < 0.0001 by ordinary 1-way ANOVA with Šídák’s multiple-comparison test.

It was previously established that human IL-33 is highly expressed in endothelial cells, namely peripheral LN HEVs (12, 14, 15), while murine IL-33 is primarily associated with epithelial cells in barrier tissues. Homology between the human *IL33* and murine *Il33* locus is poor, especially in upstream regulatory regions that, in humans, contain SNPs associated with increased prevalence of allergic asthma (16). To understand the effects of the upstream *IL33* region on expression of IL-33, we generated a “humanized” BAC transgenic mouse containing 166 kb of the human genome, including the coding region of *IL33* and the upstream regulatory regions. To prevent expression of exogenous human IL-33, the coding sequence of a Crimson reporter protein was inserted into the second exon of *IL33*, followed by a stop codon (Figure 1A). Therefore, expression of human *IL33* could be visualized by expression of Crimson. Mice containing this construct (hereafter referred to as h*IL33*^Crim^BAC mice) expressed Crimson in LN HEVs, phenocopying human expression of IL-33 in HEVs (16).

Developing the human IL-33 BAC system enabled us to compare the sources of both human and mouse IL-33 in vivo. Crimson was primarily expressed in the nonhematopoietic compartment of the lung (Supplemental Figure 1; supplemental material available online with this article; https://doi.org/10.1172/jci.insight.190418DS1). In h*IL33*^Crim^BAC mice, Crimson fluorescent reporter was observed in Lyve-1^+^ endothelium of the lung (Figure 1B), consistent with its localization throughout the vascular tree in normal human tissue (15). In m*IL33*^GFP^ mice (17), however, IL-33 reporter did not overlap with endothelial Lyve-1 staining. Rather, GFP staining was punctate and spread throughout the lung parenchyma, consistent with its reported expression in ATII cells (18) (Figure 1B). To confirm that human and murine IL-33 reporters are expressed in distinct cell types, we crossed h*IL33*^Crim^BAC mice to m*IL33*^GFP^ mice and performed immunofluorescence staining in the lungs of h*IL33*^Crim^BAC x m*IL33*^GFP^ coreporter mice. Consistent with what was seen in the parent strains, GFP primarily localized to the parenchyma and airway epithelium, while Crimson was expressed primarily in lymphatic vessels (Figure 1B). Notably, there was no overlap in Crimson and GFP expression, confirming that murine IL-33 reporter and human IL-33 reporter are expressed in distinct cell types.

In humans, IL-33 is expressed in ex vivo basal cell cultures from whole-lung human explants (19). In mice, however, basal epithelial cells are restricted to the trachea (20). As such, we stained whole tracheal mounts from h*IL33*^Crim^BAC reporter mice to determine whether Crimson hIL-33 reporter could be found in basal epithelial cells. Immunofluorescence staining of tracheas from h*IL33*^Crim^BAC mice revealed that Crimson was found throughout the submucosal glands (SMG) (Figure 1C, white arrowheads), as well as on tracheal epithelial cells lining the lumen (Figure 1C, yellow arrowheads). In both locations, Crimson colocalized with keratin 5 (Krt5), a component of the cytoskeleton of basal epithelium (20, 21). In contrast, GFP expression in the tracheas of m*IL33*^GFP^ mice was weak in the SMG (Figure 1C, white arrowheads) and completely absent from the Krt5^+^ tracheal epithelium (Figure 1C, yellow arrowheads). Thus, the 5 kb regulatory region is sufficient to impart human-specific expression of IL-33 reporter Crimson in the lung and trachea.

To define which cell types express murine IL-33 or human IL-33 reporter in the lungs, we performed flow cytometric analysis of h*IL33*^Crim^BAC x m*IL33*^GFP^ coreporter mice. The majority of Crimson^+^ cells were CD45^–^, as expected (Supplemental Figure 1, A and B). Closer inspection of CD45^–^ cells by flow cytometry (see Supplemental Figure 2 for gating strategy) revealed that Crimson (marking human IL-33) was primarily expressed in the CD31^+^ EpCAM^–^ compartment, indicating that human IL-33 is primarily expressed in endothelial, rather than epithelial, cells (Figure 1D). In contrast, the majority of GFP^+^ cells expressed EpCAM, the prototypical epithelial cell marker. Of those cells that did not express EpCAM, the majority were negative for CD31 but expressed PDPN, which identified them as lung fibroblasts (22) (Figure 1E). To validate that our human IL-33 reporter expression corresponds to *IL33* mRNA expression in humans, we compared our flow cytometry data to RNA-seq data from the Lung Gene Expression Analysis (LGEA) web portal (23). Human *IL33* mRNA was overwhelmingly expressed in endothelial cells, with little to no expression in terminally differentiated epithelium (Figure 1F), mirroring our flow cytometry findings in h*IL33*^Crim^BAC x m*IL33*^GFP^ coreporter mice. Additionally, we performed RNAscope on human lungs to visualize *IL33* transcript in lung donors without known pulmonary disease. *IL33* message could be readily visualized in the endothelium and basal epithelium throughout the lung tissue (Supplemental Figure 3). Consistent with localization of GFP in EpCAM^+^ cells of m*IL33*^GFP^ mice by flow cytometry, murine *Il33* mRNA was primarily found in sorted epithelial cells and fibroblasts, with very little expression in endothelial cells (Figure 1G). Thus, human and murine IL-33 fluorescent reporters, while both primarily expressed in nonhematopoietic cells in the lungs, localize to distinct compartments, consistent with RNAscope staining of *IL33* transcript in human lungs and data from publicly available RNA-seq datasets.

### The human IL-33 5 kb regulatory element is required for Crimson expression in the lung microvasculature of hIL33^Crim^BAC mice.

**Figure 2:**
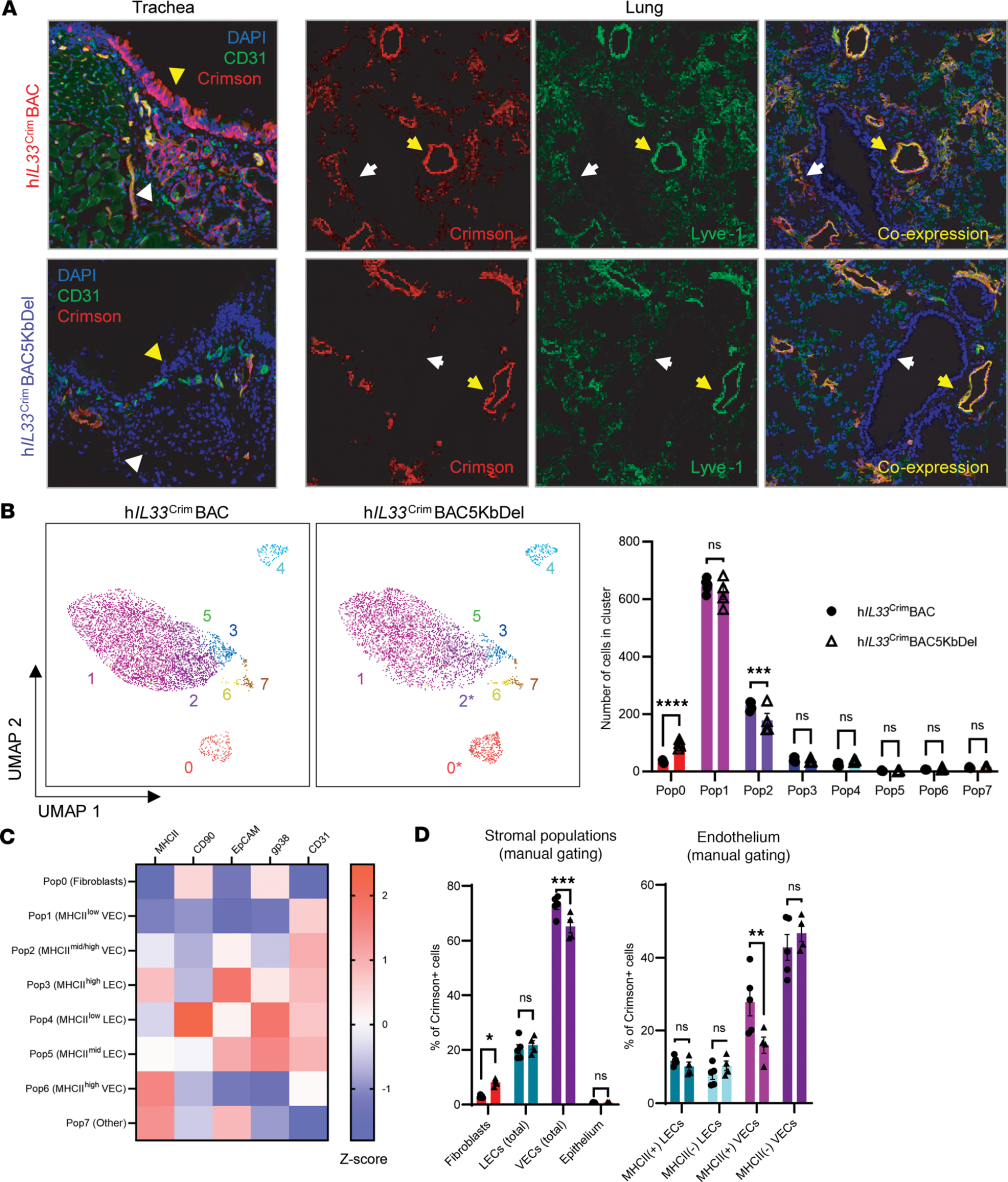
There is loss of Crimson expression in lung microvasculature of h*IL33*^Crim^BAC mice with deletion of a 5kB regulatory element. (**A**) Confocal microscopy (40×) of trachea and lung from 6- to 8-week-old naive h*IL33*^Crim^BAC reporter mice (top) or h*IL33*^Crim^BAC5KbDel reporter mice (bottom). In trachea images, yellow triangles: tracheal epithelium; white triangles: submucosal glands (SMG). In lung images, yellow arrowheads denote coexpression of Lyve-1 and Crimson in large vessels; white arrowheads denote coexpression of Lyve-1 and Crimson in the microvasculature of h*IL33*^Crim^BAC, but not h*IL33*^Crim^BAC5KbDel mice. (**B**) Left: UMAP clustering of flow cytometric data from the lungs of CD45^–^Crimson^+^ cells from h*IL33*^Crim^BAC and h*IL33*^Crim^BAC5KbDel mice. Each color represents a unique cluster generated by FlowSOM. All cells are concatenated from *n* ≥ 4 mice per group, with 1,000 Crimson^+^ cells downsampled from each mouse. Right: Quantification of cells within each FlowSOM cluster per sample. (**C**) Heatmap of stromal cell marker median fluorescence intensity (MFI) within each FlowSOM cluster, generated using the ClusterExplorer plugin. (**D**) Left: quantification of fibroblasts, LECs, VECs, and epithelial cells positive for Crimson (h*IL33* reporter) using the manual gating strategy in Supplemental Figure 2. Right: breakdown of Crimson^+^ LECs and VECs by expression of MHCII. Data from **B**–**D** are from a single experiment, with *n* ≥ 4 mice per genotype. Quantified data are represented as mean ± SEM. *P < 0.05; **P < 0.01; ***P < 0.001; ****P < 0.0001 by 2-way ANOVA with Šídák’s multiple-comparison test.

To test the importance of the 5 kb regulatory region for expression of human *IL33*, we generated a modified h*IL33*^Crim^BAC mouse that does not contain the 5 kb SNP-containing region we described previously (16) (h*IL33*^Crim^BAC5KbDel mice). These mice do not contain Crimson expression in the LN HEVs, highlighting the importance of this region in controlling LN expression of human IL-33 (16). Consistent with its importance in controlling *IL33* expression in LNs, loss of the 5 kb regulatory region in h*IL33*^Crim^BAC5KbDel completely abrogated expression of Crimson in tracheal basal epithelium and cells within the SMG (Figure 2A).

To our surprise, Crimson expression in large endothelial vessels remained intact in the lungs of both h*IL33*^Crim^BAC and h*IL33*^Crim^BAC5KbDel mice (Figure 2A, yellow arrowheads), suggesting that larger vessels may express *IL33* independently of the 5 kb regulatory region. However, smaller vessels positive for Crimson in the h*IL33*^Crim^BAC mouse lung were negative for Crimson in the h*IL33*^Crim^BAC5KbDel lungs (Figure 2A, white arrowheads). Thus, the 5 kb regulatory region upstream of Crimson (our human IL-33 reporter) is dispensable for IL-33 reporter expression in major endothelial vessels but is required for its expression in smaller endothelial vessels.

To understand how the 5 kb region upstream of the human IL-33 gene affects its expression in specific lung structural cells, we turned toward flow cytometric analysis of Crimson-expressing cells from the lungs of h*IL33*^Crim^BAC and h*IL33*^Crim^BAC5KbDel mice. Unsupervised clustering analysis of Crimson^+^ cells from both strains generated 8 distinct clusters of Crimson-expressing cells (Figure 2B). When visualized by UMAP, the second-most prevalent cluster of Crimson-expressing cells were significantly reduced in h*IL33*^Crim^BAC5KbDel mice relative to h*IL33*^Crim^BAC mice (Figure 2B, orange cluster). Closer investigation of this cluster using the ClusterExplorer plugin in FlowJo revealed that these cells were endothelial (CD31^hi^) but negative for PDPN, consistent with their identity as vascular endothelial cells (VECs) (Figure 2C). Additionally, these cells express high levels of MHCII, which is notable as previous work identified *H2-Ab1* as a core marker in a subset of lung capillary cells (24). Manual gating of MHCII^hi^ VECs confirmed that these cells express more h*IL33* reporter in h*IL33*^Crim^BAC mice than the corresponding cells in h*IL33*^Crim^BAC5KbDel mouse lungs (Figure 2D). These results demonstrate that the 5 kb regulatory region we identified in the human *IL33* locus is important for constitutive Crimson expression in LECs of the LN, basal epithelium and SMG of the trachea, and microvasculature in the lung.

The 5 kb GWAS-associated region within the h*IL33*^BAC^Crim construct contains several transcription factor (TF) binding sites that are not conserved in the corresponding murine region upstream of *Il33* (16). The dependence of microvasculature — but not larger vessels — on the 5 kb region for expression of hIL33 reporter could be due to differential TF expression in endothelial cells from these subsets. To address this possibility, we compiled a list of TFs with demonstrated binding sites in the 5 kb regulatory region using the UCSC Genome Browser (25). We then compared expression of these TFs across stromal cell compartments in a previously annotated human lung scRNA-seq dataset (26) and murine lung scRNA-seq dataset (27). The expression of most TFs did not differ dramatically between capillaries and larger blood vessels in either human or murine lungs (Supplemental Figure 4, A and B; compare columns 4 and 5 versus 7 and 8). *FOS*, which encodes a component of the AP-1 TF complex, was expressed in a lower proportion of capillary endothelial cells than vascular arterial/venous cells, mimicking the expression pattern of *IL33* in both compartments. Mice also had lower relative expression of the orthologous *Fos* in microvasculature (aCap and gCAP) than in larger vessels (Arterial Endo, Venous Endo). While these trends certainly suggest that differential TF usage may affect total expression of *IL33*, other factors, such as differential chromatin accessibility, alternative promoter usage, and even posttranscriptional regulatory mechanisms could also contribute to the sensitivity of microvasculature to 5 kb regulatory region-driven *IL33* expression.

### Human IL-33 reporter is downregulated in the lung following sensitization and challenge with HDM allergen.

**Figure 3:**
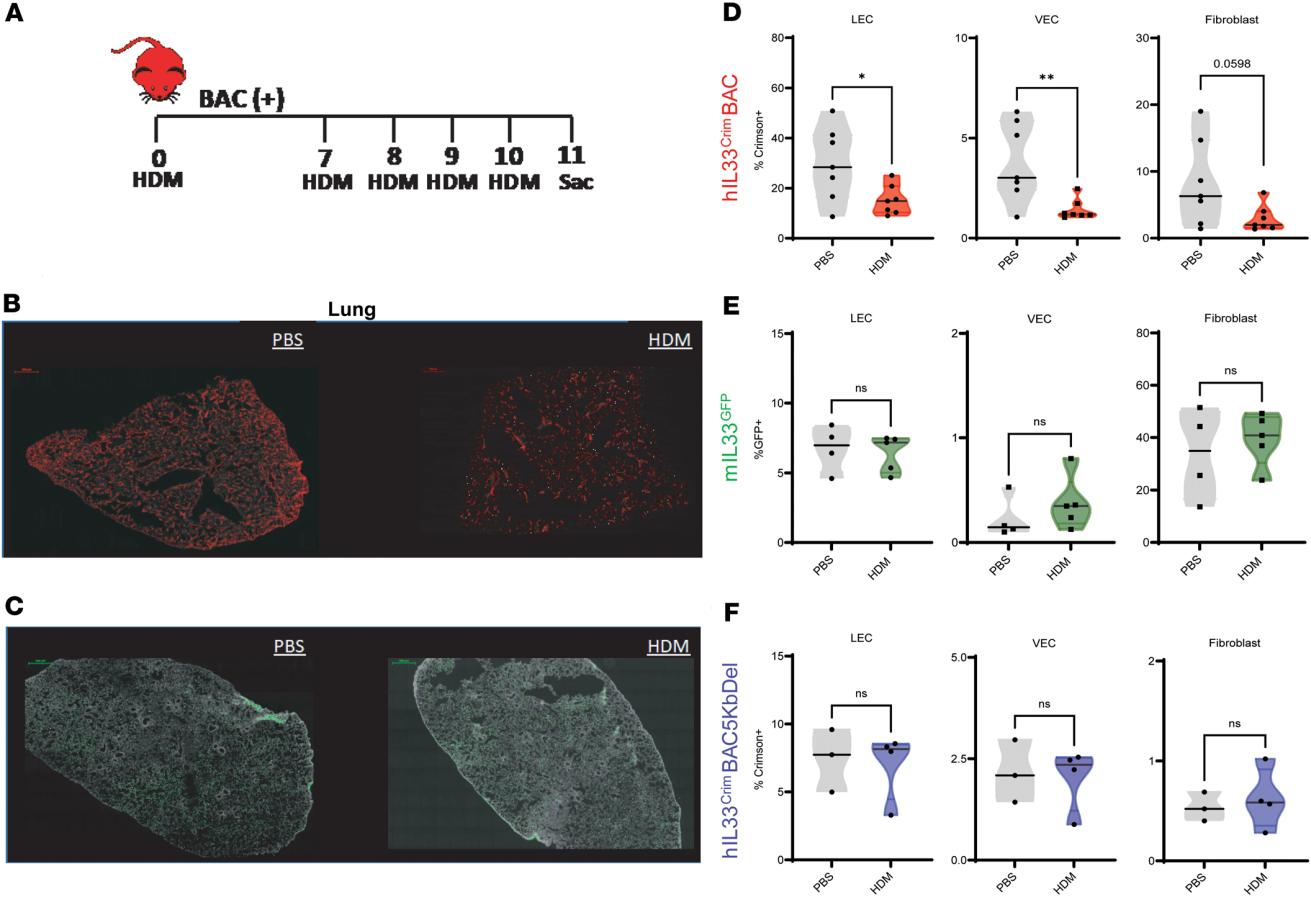
*hIL33* reporter is downregulated in the lung following intratracheal administration of house dust mite (HDM) extract. (**A**) Schematic describing HDM extract treatment in h*IL33*^Crim^BAC (BAC^+^) mice. Mice received 50 μg HDM extract on day 0 and 25 μg HDM extract on days 7–10. Control mice received 50 μL PBS at days 0 and 7–10. (**B**) Confocal microscopy of lungs from h*IL33*^Crim^BAC mice treated with PBS (left) or HDM (right). (**C**) Confocal microscopy of lungs from m*IL33*^GFP^ mice treated as in **A**. Scale bars: 100 μm. (**D**) Percentage of LECs, VECs, and fibroblasts expressing Crimson in h*IL33*^Crim^BAC mice treated as in **A**. (**E**) Percentage of LECs, VECs, and fibroblasts expressing mIL33-GFP in m*IL33*^GFP^ mice treated as in **A**. (**F**) Percentage of LECs, VECs, and fibroblasts expressing Crimson in h*IL33*^Crim^BAC5KbDel mice treated as in **A**. Data from **D** are from 2 independent experiments, with *n* ≥ 3 mice per group. Data from **E** depict a single representative experiment (of 2 experiments) with *n* ≥ 3 mice per group. Data from **F** depict a single experiment with *n* ≥ 4 mice per group. Quantifications in **D**–**F** are represented as mean ± first and third quartile. *P < 0.05; **P < 0.01 by unpaired *t* test. Scale bars: 500 μm.

Extracts from the common environmental allergen HDM have been shown to elicit potent IL-33–dependent responses in mice (28–30). Thus, to examine the dynamics of human IL-33 reporter expression in response to allergen, we sensitized and challenged h*IL33*^Crim^BAC mice with HDM or PBS (Figure 3A). At day 11, lungs were harvested for quantification of Crimson (*IL33* reporter) expression by immunofluorescence and flow cytometry. Surprisingly, HDM sensitization and challenge resulted in a reduction in Crimson^+^ area over the whole lung relative to mice given PBS (Figure 3B). Closer analysis of stromal cells in h*IL33*^Crim^BAC mice revealed that Crimson expression was downregulated in CD31^+^PDPN^+^ lymphatic endothelial cells (LECs), CD31^+^PDPN^–^ VECs, and to a lesser extent CD31^–^ fibroblasts (Figure 3D), while Crimson expression in EpCAM^+^ epithelial cells remained unchanged (Supplemental Figure 5A). While the lung endothelium in h*IL33*^Crim^BAC mice responded to allergen challenge by downregulating h*IL33* reporter, no observable change in GFP was found in lung endothelial compartments of m*IL33*^GFP^ mice after allergen sensitization (Figure 3, C and E). Thus, murine epithelium and human endothelium may represent reciprocal sources of IL-33 in each species, but only lung endothelium directly responds to allergen exposure by downregulating IL-33.

To determine whether the 5 kb region was required for the downregulation of the human IL-33 reporter, we sensitized and challenged h*IL33*^Crim^BAC5KbDel mice with HDM. Crimson expression was not significantly altered in either lung VECs or LECs (Figure 3F), despite comparable levels of allergic airway inflammation with h*IL33*^Crim^BAC mice (data not shown). This regulatory region, therefore, not only governs human IL-33 reporter expression at baseline, but it is also, surprisingly, necessary for its downregulation in lung endothelial cells following allergen challenge.

### Crimson expression is reduced in pulmonary vasculature after murine IL-33 administration in an ST2-dependent manner.

**Figure 4:**
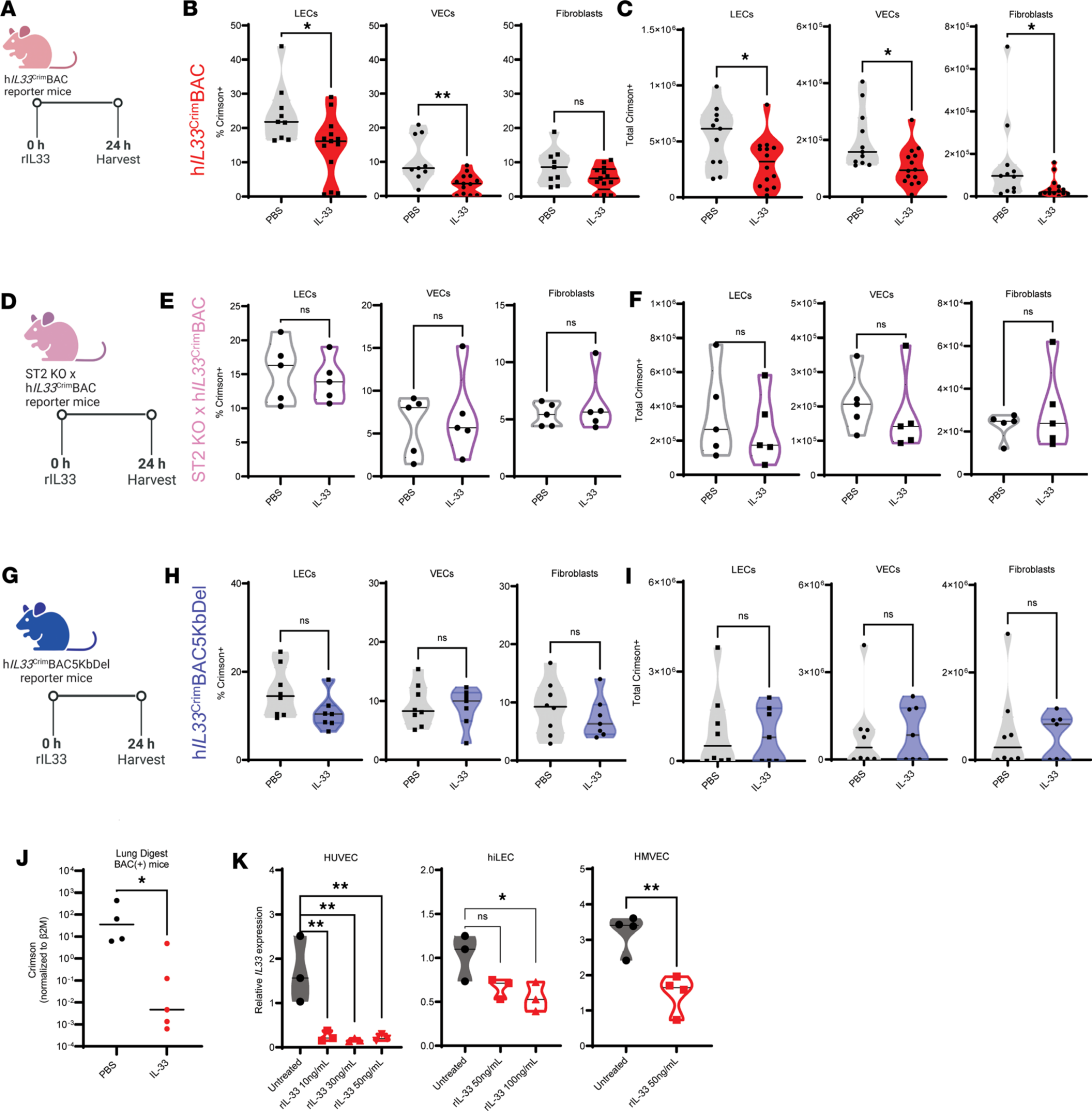
Crimson expression is reduced in pulmonary vasculature after murine IL-33 administration in an ST2-dependent manner. (**A**) Schematic describing intratracheal treatment of h*IL33*^Crim^BAC mice with 100 ng of recombinant IL-33 (rIL33). Crimson fluorescence was measured in lung stromal cells 24 hours later. (**B**) Crimson expression in CD45^–^ cells from the lungs of h*IL33*^Crim^BAC mice treated with PBS or rIL33, expressed as percentage of cells positive for Crimson. (**C**) Crimson^+^ cells in the lungs of h*IL33*^Crim^BAC mice treated with PBS or rIL33, quantified as total backcalculated number of Crimson^+^ cells. (**D**) Schematic describing i.t. treatment of ST2 KO x h*IL33*^Crim^BAC mice with PBS or 100 ng rIL33. (**E**) Crimson expression in CD45^–^ cells from the lungs of ST2 KO x h*IL33*^Crim^BAC mice treated as in **D**. (**F**) Crimson^+^ cells in the lungs of ST2 KO x h*IL33*^Crim^BAC mice treated with PBS or rIL33, quantified as total backcalculated number of Crimson^+^ cells. (**G**) Schematic describing i.t. treatment of h*IL33*^Crim^BAC5KbDel mice with PBS or 100 ng rIL33. (**H**) Crimson expression in CD45^–^ cells from the lungs of h*IL33*^Crim^BAC5KbDel mice treated as in **G**. (**I**) Crimson^+^ cells in the lungs of h*IL33*^Crim^BAC5KbDel mice treated with PBS or rIL33, quantified as total backcalculated number of Crimson^+^ cells. (**J**) *Crimson* transcript levels in total lung digests from h*IL33*^Crim^BAC mice treated intratracheally with PBS or rIL33 as in **A**. (**K**) Quantification of *IL33* transcripts in HUVECs, hiLECs, and HMVECs 24 hours after treatment with the indicated dose of rIL33. Data in **A**–**I** are from 2 independent experiments with *n* ≥ 3 samples per treatment. Data in **J** are from a single representative experiment. Data for each cell line in **K** are from a single experiment with *n* ≥ 3 technical replicates. Data in **B**–**F** and **K** are represented as mean ± first and third quartile; data in **J** depict all individual values with a dotted line at the mean. *P < 0.05; **P < 0.01 by unpaired *t* test.

Administration of complex allergens such as HDM in the lung results in release of intracellular IL-33 into the extracellular space (31), activating local innate immune cells such as ILC2s, DCs, and Th2 cells (32). We hypothesized that downregulation of Crimson reporter after allergen administration was due to a negative feedback loop, in which extracellular IL-33 prevented generation of de novo human IL-33 transcripts. To test this hypothesis, we administered 100 ng recombinant murine IL-33 intratracheally to h*IL33*^Crim^BAC mice and, 24 hours later, measured Crimson fluorescence in the stromal cells of these mice (Figure 4A). Remarkably, a single dose of IL-33 was sufficient to downregulate Crimson expression in the lung endothelium of h*IL33*^Crim^BAC mice, both by percentage of Crimson^+^ endothelial cells (Figure 4B) and total number of Crimson^+^ endothelial cells (Figure 4C). To address whether this phenomenon was dependent on sensing of IL-33 through its cognate receptor ST2, we crossed h*IL33*^Crim^BAC mice onto an ST2-deficient background and administered recombinant IL-33 twenty-four hours before measuring Crimson expression in the lung endothelium (Figure 4D). Unlike h*IL33*^Crim^BAC mice, ST2KO x h*IL33*^Crim^BAC mice did not downregulate Crimson expression in LECs, VECs, or fibroblasts by either percentage or absolute number (Figure 4, E and F). Similar results were obtained in h*IL33*^Crim^BAC5KbDel mice (Figure 4, G–I) and m*IL33*^GFP^ mice (data not shown) given IL-33, indicating that IL-33–dependent downregulation of IL-33 reporter depends both on IL-33 sensing by ST2 and presence of the 5 kb upstream regulatory region.

### Exogenous IL-33 downregulates IL-33 transcript levels in human endothelial cells.

We sought to determine whether IL-33 treatment affects human IL-33 reporter expression at the level of RNA expression. Whole-lung digests of mice treated with recombinant IL-33 contained fewer Crimson transcripts than mice that received PBS alone, indicating that Crimson was being downregulated at the transcriptional level (Figure 4J). To assess whether the downregulation of *IL33* transcripts also occurred in human cells, we turned to in vitro cultures of various human endothelial cells, which include human umbilical vein endothelial cells (HUVEC), human immortalized LECs (hiLEC), and human lung micro–vascular endothelial cells (HMVECL). After 24 hours, recombinant IL-33 downregulated human *IL33* transcript abundance in all 3 endothelial cell lines, with doses as low as 10 ng/mL (Figure 4K). Thus, IL-33–dependent downregulation of IL-33 reporter in h*IL33*^Crim^BAC lung endothelium faithfully recapitulated IL-33–dependent downregulation of *IL33* in multiple human-derived endothelial cell lines.

## Discussion

While animal models are valuable for dissecting human disease mechanisms, the degree to which they capture the full complexity of the human immune system remains limited. For example, the coding sequence of IL-33 shares only 52% amino acid sequence homology between mice and humans, with even less homology in noncoding regulatory regions (12). Consistent with this lack of homology, human and murine IL-33 are expressed in different cell types of the lung; murine *Il33* is expressed in type 2 alveolar epithelium and is largely absent from the lung vascular network (13, 18), while human IL-33 is found mostly in basal epithelium and endothelium (5, 12). Using our human IL-33 reporter transgenic system, we have identified a negative feedback loop reliant on a regulatory region just upstream of the human *IL33* gene (16) that is necessary for human *IL33* reporter expression in the microvasculature. Excitingly, the expression of Crimson reporter protein and transcript in our system recapitulates the localization of human *IL33* mRNA in the lung cells of publicly available RNA-seq datasets and our own staining for *IL33* transcript in human lungs. Furthermore, we identified a negative feedback loop in which exogenous IL-33 reduces human *IL33* reporter transcript in h*IL33*^Crim^BAC mice or human *IL33* mRNA in endothelial cell lines.

As murine IL-33 is found abundantly in lung ATII cells, most IL-33 studies in humans have described *IL33* expression in bronchoalveolar lavages (BAL), nasal epithelial brushings, or epithelial cell lines (6, 13, 33). These studies have demonstrated that epithelial cells from patients with barrier dysfunction — most notably, allergic asthma — upregulate *IL33* relative to controls. However, a direct comparison of human epithelial and endothelial *IL33* expression has been difficult in living tissue due to the paucity of endothelial cells in epithelial cell brushings (34). scRNA-seq analyses of human lungs have demonstrated that micro–vascular endothelial cells and other endothelial cell subsets express higher levels of *IL33* transcript than either AT1 or AT2 cells (Supplemental Figure 4). In this study, we have confirmed using our h*IL33*^Crim^BAC reporter mice and human cell lines that the lung endothelium is a major contributor to lung IL-33 levels, but we recognize that the bronchial epithelium also contributes, especially in asthmatic patients.

Our work does not address the relative contribution of *IL33* from the basal epithelium and endothelium in the same model system, as murine lungs contain very few basal epithelial cells outside of the trachea (20). These differences between mice and humans make it difficult to study epithelial *IL33* transcript levels in our h*IL33*^Crim^BAC mice. However, we have demonstrated that tracheal basal epithelium from h*IL33*^Crim^BAC reporter mice also express human IL-33 reporter. Due to the low expression of *IL1RL1* — the gene encoding the IL-33 receptor ST2 — in basal epithelium from human cells (data not shown), we anticipate that basal epithelium does not respond to extracellular IL-33 by downregulating *IL33* transcript. This hypothesis is corroborated by a recent publication which found that human basal epithelium cocultured with IL-33 did not significantly induce up- or downregulation of the IL-33 splice variant *IL33B* (35). Furthermore, another study demonstrated that IL-33 production in human basal epithelial cells is “unleashed” by mast cell recruitment and secretion of type 2 cytokines (36).

The vascular network in the human body forms a critical component of regulation in health and disease. Here, our data demonstrate that a 5′ IL-33 regulatory element imparts the ability of the endothelium to respond to extracellular IL-33 by modulating total *IL33* mRNA. Specifically, pulmonary endothelial cells respond to extracellular IL-33 engagement with ST2 by downregulating *IL33* transcript levels. This downregulation appears to be unique to the endothelial compartment, as a recent study by Nawijn and colleagues failed to observe IL-33–induced changes in the transcriptome of primary cultured bronchial epithelial cells (37). In another study, Altman and colleagues observed that both HDM and exogenous IL-33 increased the expression of *IL33* in differentiated epithelial cells in vitro (36). In our model, we did not observe an increase in epithelial Crimson reporter after i.t. instillation of HDM or exogenous IL-33. The upregulation of IL-33 may rely on regions other than the 5 kb region, or there may be other unappreciated pharmacologic differences between humans and mice regarding IL-33–ST2 ligand-receptor interactions.

In light of these findings, why would endothelial cells — which constitutively express IL-33 — downregulate *IL33*, and how does this downregulation affect allergic asthma? In mice and humans, IL-33 protein is primarily stored in the nucleus; therefore, it is possible that IL-33 protein itself is acting as a transcriptional repressor, as previously reported (14). However, a subsequent study found that endothelial cells with RNAi-mediated silencing of endogenous nuclear IL-33 had no detectable changes in the proteome relative to endothelial cells transfected with control siRNA (38). Therefore, it is unlikely that IL-33 protein — exogenous or endogenous — is modulating transcription by binding directly to chromatin to repress transcription. Rather, modules downstream of the IL-33/ST2 signaling complex are likely responsible for reducing intracellular *IL33* expression, as loss of ST2 was sufficient to prevent IL33-induced downregulation of *IL33* reporter in endothelial cells.

Within the 5 kb regulatory element we defined in our previous work (16) are binding sites for TFs such as FOS and GATA3, both of which are activated after IL-33 engagement with ST2 (39). Additionally, *FOS* is highly expressed in endothelial cells of the lung and basal epithelium relative to terminally differentiated AT2 cells in humans (Supplemental Figure 4A). This raises the possibility that IL-33/ST2 engagement in endothelial cells results in TF binding to the 5 kb regulatory element, thereby repressing *IL33* transcription and preventing overexpression of *IL33* when extracellular IL-33 is present. This model would be particularly compelling in the context of genetic variants within the 5 kb regulatory region, as we have already demonstrated that asthma-associated SNP rs1888909 engages with the transcriptional repressor OCT-1 (16). However, differential TF binding to the 5 kb regulatory region does not explain why its loss abolishes *IL33* reporter expression in the microvasculature of h*IL33*^Crim^BAC5KbDel mice. We envision that the molecular players responsible for downregulating *IL33* upon sensing of extracellular IL-33 are distinct from those that allow expression of *IL33* in the endothelial and basal epithelial compartments at baseline.

A second possibility is that IL-33/ST2 signaling negatively regulates *IL33* transcript abundance at the posttranscriptional level. microRNA-mediated (miRNA-mediated) silencing of *Il33* has already been observed in multiple murine organ systems such as liver (40) and lung (41, 42). The role of miRNAs in posttranscriptional regulation of human *IL33* transcript is less well studied. In the context of allergic asthma, miR-200b/c is underrepresented in BAL cells from human asthmatics and has been demonstrated to bind to the IL-33 3′ UTR in Jurkat cells (43). It can be imagined that receptor:receptor ligand interactions, such as IL-33 engagement with ST2, could induce expression of miRNAs such as miR-200b/c and silence *IL33* transcripts in the nucleus and cytosol. Whether this occurs in human lung endothelium, and whether asthma-associated SNPs affect the binding and function of miRNAs, remain interesting open questions.

IL-33–mediated downregulation of endothelial *IL33* expression provides an interesting potential mechanism by which endothelial cells maintain lung homeostasis. If engagement of extracellular IL-33 with ST2 represses *IL33* expression, then depletion of extracellular IL-33 may be sensed directly by the endothelium, which in turn responds by producing *IL33*. Therefore, pharmacological inhibition of IL-33 by antibodies such as itepekimab may not effectively block IL-33–mediated inflammation. Rather, the paucity of IL-33 in the extracellular space may prompt the endothelium to produce more *IL33*, thereby canceling out the effects of IL-33 blockade. Importantly, this phenomenon may not have been observed in human studies, as epithelial cells do not respond in the same way to exogenous IL-33 but would be one of a few cell subsets available to sample by BAL or bronchial brushings. Therefore, our BAC transgenic system allowed us to uncover an important self-regulating circuit of IL-33 production in humans and underscores the importance of noncoding elements in dynamic responses to extracellular cytokine levels.

## Methods

### Sex as a biological variable.

All studies used male and female mice, as there was no observed difference in responses by sex.

### Experimental animals.

C57BL/6 (B6) mice were purchased from Harlan Industries. B6.ST2^–/–^ mice were provided by A. McKenzie (Medical Research Laboratory, University of Cambridge, Cambridge, UK). IL-33^–/–^ mice were generously provided by P.J. Bryce (Northwestern University, Chicago, Illinois, USA) vis-a-vis Dirk Smith (Amgen, Thousand Oaks, California, USA) and by H.A. Turnquist (University of Pittsburgh, Pittsburgh, Pennsylvania, USA) through S. Nakae (University of Tokyo, Tokyo, Japan). For the h*IL33*^Crim^BAC mice, a BAC clone (RP11-725F15) was purchased from the NCBI Clone Registry and modified to include an E2-Crimson (Takara Biosciences) reporter gene at ATG start codon in exon 2 using standard recombineering techniques. A stop codon was inserted after the fluorescent reporter. The h*IL33*^Crim^BAC5KbDel mice is identical to the full-length construct, but with the removal of the 5 kb region at position Chr9: 6194500-6199500. Generation of h*IL33*^Crim^BAC and h*IL33*^Crim^5KbDel BAC transgenic mice was performed by the University of Chicago Transgenic Core Facility (Chicago, Illinois, USA). Briefly, modified DNA was diluted to a concentration of 2 ng/μL and used for pronuclear AIS injections of CD1 embryos in accordance with standard protocols approved by the University of Chicago. The full-length construct in the BAC was able to recapitulate *IL33* expression in 5 separate founder lines by qPCR. Founders were backcrossed to B6 mice for *n* > 10 generations. All mice were bred and housed in specific pathogen–free facilities maintained by the University of Chicago Animal Resources Center.

### Animal treatment and preparation of tissues for flow cytometry.

HDM (XPB82D3A25, Stallergenes Greer) in sterile, endotoxin-free phosphate-buffered saline (PBS) at a final volume of 50 μL/mouse was administered to mice i.t. on day 0 with 50 μg of HDM and again on day 7, 8, 9, and 10 with 25 μg HDM, before being euthanized 24 hours after the last challenge. In some experiments, mice received 100 ng of recombinant murine IL-33 (580506, BioLegend) in 50 μL of sterile, endotoxin-free PBS 24 hours before harvest. At sacrifice, lungs were dissected away from the trachea and dissociated by mechanical mincing followed by digestion with 600 U/mL Collagenase IV (C-5138, Sigma-Aldrich) and 20 μg/mL of DNase I (LS002138, Worthington Biochemical Corporation) in 10 mL for 60 minutes. Samples were then disrupted via pipetting and filtered, before RBCs were lysed using ammonium-chloride-potassium (ACK) lysis buffer for 1 minute. After RBC lysis, cells were washed and resuspended in DMEM (11995065, Thermo Fisher Scientific) containing 5% fetal calf serum (X&Y Cell Culture), MEM nonessential amino acids (11140050, Thermo Fisher Scientific), and HEPES (15630080, Thermo Fisher Scientific). For flow cytometric analysis, 0.5 × 10^6^ to 1 × 10^6^ cells were aliquoted to 5 mL polypropylene tubes. For some studies, cells were washed with 1× PBS and stained with Zombie Aqua viability dye (423102, BioLegend) at a 1:1,000 dilution. Ten μL of anti-CD16/32 (clone 2.4G2) was used to block Fc receptors to prevent nonspecific binding of fluorescent antibodies. Fluorescently conjugated antibodies used for flow cytometry are detailed in Table 1.

After staining, cells were washed with PBS containing 0.1% sodium azide and 0.2% bovine serum albumin and then fixed overnight in 2% methanol-free formaldehyde solution (28906, Thermo Fisher Scientific). All data were collected using a BD Biosciences LSRFortessa and analyzed using FlowJo software (Tree Star Inc.). All instruments were maintained by the University of Chicago Flow Cytometry and Antibody Technology Core Facility and the University of Virginia Flow Cytometry Core Facility.

### Tissue preparation and immunofluorescence staining.

Mouse lungs and trachea were fixed upon removal in 2% methanol-free formaldehyde solution for 90 minutes at room temperature, washed in 1× PBS, and then placed in 30% sucrose solution. The solution was changed 2–3 times over a period of 24 hours, after which lobes were allowed to sit in the sucrose until they sank in the tube. After sinking to the bottom of the sucrose solution, lungs and tracheas were embedded in Optimal Cutting Temperature compound (OCT 4583, Sakura Finetech) and flash-frozen with liquid nitrogen. Blocks were stored at –80°C until sectioning. Frozen tissues were sliced into sections 5–7 μm thick and dried onto Superfrost Plus slides (Thermo Fisher Scientific) overnight. After drying, tissue sections were permeabilized with 0.5% IGEPAL CA-630 (Sigma-Aldrich) for 5 minutes at room temperature, before being quenched and blocked with 10% normal goat serum (NGS; Sigma-Aldrich) in 50 mM NH_4_Cl. Tissues were immunostained with primary antibodies including Living Colors DsRed Polyclonal anti-E2 Crimson (Clontech [now Takara Bio]); anti-mouse CD45 (clone 30-F11) and anti-mouse KRT5 (clone poly9059) (both from BioLegend); anti-mouse Lyve-1 (Affymetrix/eBioscience clone ALY7) and anti-mouse CD31 (Life Technologies clone 2H8) (both from Thermo Fisher Scientific); and polyclonal anti-GFP:FITC (Novus Biologicals). Sections were washed and stained with secondary antibodies including goat anti-rabbit IgG:Alexa Fluor 633 and goat anti-rat IgG:Alexa Fluor 680 (both from Life Technologies, Thermo Fisher Scientific); goat anti-Armenian hamster IgG:AlexFluor 568 and goat anti-chicken IgY:AlexFluor 680 (both from Abcam); and nucleic acid stain Hoechst 33342 (Life Technologies, Thermo Fisher). All antibody mixes were prepared in wash buffer containing 10% NGS and 1× PBS. After washing, coverslips (Thermo Fisher Scientific) were set with ProLong Diamond Antifade Mountant (Life Technologies, Thermo Fisher) and allowed to cure overnight before being sealed.

### Imaging.

Imaging was performed at the University of Chicago Integrated Light Microscopy Facility. Images were captured with a Leica SP8 laser scanning confocal microscope (Leica Microsystems Inc.) using a 20×/0.7 multi-immersion objective and LAS_X Leica acquisition software. Further processing of images was completed using ImageJ software (NIH).

### Cell culture, cells, and reagents.

Human umbilical vein-derived endothelial cells (HUVECs; C2519A, Lonza) and cultured in EBM Basal Medium (CC-3156, Lonza) and EGM-2 SingleQuots Supplements (CC-4176, Lonza). Human immortalized dermal LECs (hiLECs) were a gift from Melody Swartz at the University of Chicago’s Pritzker School of Molecular Engineering. Human lung microvascular endothelial cells (HMVEC-L) were purchased from Lonza (catalog CC-2527) and cultured in EBM Basal Medium and EGM-2 MV Microvascular Endothelial Cell Growth Medium SingleQuots supplements (catalog CC-4147, Lonza). Cells were maintained at 37°C in 95% humidity/5% CO_2_ atmosphere and were split at a ratio of 1:3 before reaching confluence. The cultures were used at passage level 1–5. All experiments were performed on confluent cultures. Upon reaching confluence, cells were plated at 2 × 10^4^/mL in a 24-well plate and treated with varying concentrations of recombinant IL-33 (BioLegend).

### qPCR.

Total cellular RNA was extracted using the Quick-RNA MicroPrep Kit (R1051, ZymoResearch) and RNA was reverse-transcribed with Applied Biosystems High-Capacity cDNA Reverse Transcription Kit (4368814, Thermo Fisher Scientific). For qPCR, a total volume of 25 μL containing 1 μL cDNA template, 0.5 μM of each primer (Table 2), and SYBR Green PCR Master Mix (Applied Biosystems) was analyzed in quadruplicate. Gene expression was analyzed with an ABI PRISM 7300 Sequence Detector and ABI Prism Sequence Detection Software version 1.9.1 (Applied Biosystems). Results were normalized by division of the value for the unknown gene by that obtained for housekeeping gene GAPDH or B2m.

### RNAscope.

RNA in situ hybridization technology (RNAscope) was used to hybridize IL-33 mRNA probes (catalog 400118-C2) and customized from ACD using the RNAscope 2.5 LS Duplex Reagent Kit (catalog 322440) onto at least 7 sections from lung samples from 10 donors by the Human Tissue Research Center (HTRC) of the University of Chicago. Nontransplantable control lungs were obtained from the Gift of Hope/Regional Organ and Tissue Donor Network organ and tissue donor network, an organ procurement organization that provides services regionally to 12 million people within the national donation system. Patients from whom control specimens (GOH) were obtained had no known history of lung disease or immunologic disorder. Lung tissue was taken from left upper lobe, formalin-fixed, and paraffin-embedded for use. H&E staining to confirm asthma diagnosis and identify anatomical landmarks was performed on adjacent sections.

### TF mRNA quantification in stromal cells within scRNA-seq datasets.

A list of TFs that bind within the 5 kb regulatory region upstream of human *IL33* was generated using the UCSC Genome Browser (25). Human lung scRNA-seq data was from Madissoon et al. (26) (10 donors, 193,108 cells) available at https://5locationslung.cellgeni.sanger.ac.uk/ The dataset was viewed using BBrowserX Pro (BioTuring) using the author’s cell annotations, with manual concatenation of the “systemic vascular” and “pulmonary vascular” groups into a single “vascular” group for both arterial and venous cells. Plots were generated using BBrowserXPro with log normalized expression and relative scaling. The murine scRNA-seq dataset was from Curras-Alonso et al. (27) (5 control mice, 28,779 cells) available at https://www.ncbi.nlm.nih.gov/geo/query/acc.cgi?acc=GSE211713 Murine homologs/orthologs of human genes were used to manually generate equivalent cell type groups to those in Madissoon et al. (26) with additional annotation of the basal epithelium from Plasschaert et al. (44). Plot for murine scRNA-seq dataset was generated using BBrowserX Pro as described above.

### Statistics.

All statistical analyses were performed with GraphPad Prism software, and *P* values less than 0.05 were considered significant. For comparisons of 2 groups, an unpaired Student’s 2-tailed *t* test (parametric) or Mann-Whitney *U* test (nonparametric) was performed. To determine whether data were parametric or nonparametric, variances were compared using an *F* test. For comparisons of 3 or more groups, a 2-way ANOVA with Šídák’s multiple comparisons post hoc test was conducted. Data are plotted as mean ± SEM.

### Study approval.

All studies conformed to the principles set forth by the Animal Welfare Act and the NIH guidelines for the care and use of animals in biomedical research. The experiments were approved by the University of Chicago IACUC and University of Virginia IACUC.

### Data availability.

All values corresponding to Figures 1–4 and Supplemental Figures 1–5 are available in the Supporting Data file. Raw data for Supplemental Figure 4A is available on the European Nucleotide Archive (ENA) under Project PRJEB52292 and BioStudies accession S-SUBS17. Raw data for Supplemental Figure 4B is available in the Gene Expression Omnibus (GEO) under accession no. GSE211713.

## Author contributions

MKH, CL Howard, DCD, NS, IA, MAN, and AIS conceived and designed the study. MKH, CL Howard, DCD, KMB, DFC, EMG, CL Hrusch, RSG, and FAO conducted experiments, acquired, and analyzed data. RTH and JMS analyzed scRNA-seq datasets for Supplemental Figure 4. PAV, TEV, and MAN contributed valuable insight for data interpretation and analysis. MKH, CL Howard, DCD, and AIS wrote the manuscript. All authors read, edited, and approved the final manuscript. MKH and CL Howard share first authorship of this work. CL Howard generated initial findings, wrote a first draft of the manuscript, and left the institution prior to submission; MKH repeated and extended these findings, reanalyzed data, and wrote the submitted manuscript. The first author listed was decided by amount of work performed.

## Conflict of interest

The authors have declared that no conflict of interest exists.

## Funding support

This work is the result of NIH funding, in whole or in part, and is subject to the NIH Public Access Policy. Through acceptance of this federal funding, the NIH has been given a right to make the work publicly available in PubMed Central.

- NIH grant R01HL118758 (to AIS and MAN)
- NIH grant U19AI162310 (to AIS and MAN)
- NIH grant R01AI125644 (to AIS)
- NIH grant F31HL156659 (to MKH)
- NIH grant R01AI125644-05S1 (to AIS and MKH)
- São Paulo Research Foundation FAPESP, 2024/13576-2 (to FAO)

## Supplementary Material

Supplemental data

Supporting data values

## Figures and Tables

**Table 1:**
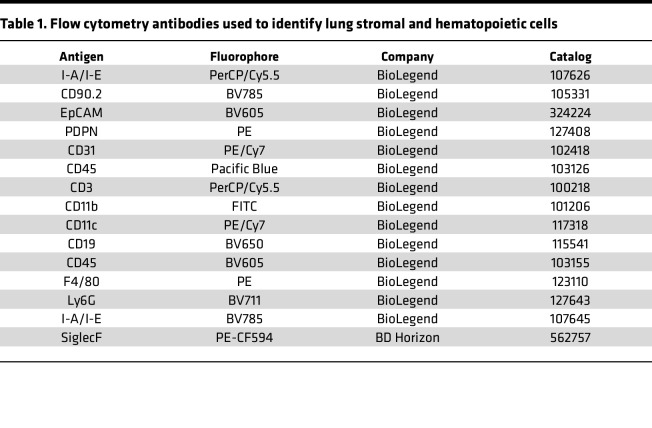
Flow cytometry antibodies used to identify lung stromal and hematopoietic cells

**Table 2:**
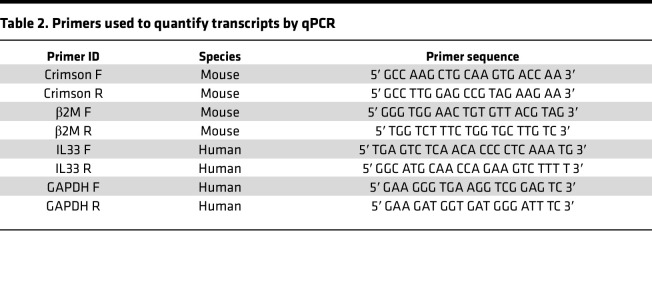
Primers used to quantify transcripts by qPCR

## Version 1 05/08/2026

Electronic publication
