# Supplementary material for: Negative regulation of human IL-33 in endothelium during allergic airway inflammation
Source: JCI Insight. 2026 May 8;11(9):e190418. doi: 10.1172/jci.insight.190418 (PMC13232004; doi:10.1172/jci.insight.190418)
Supplement: Supplemental data [file jciinsight-11-190418-s087.pdf]

Negative regulation of human IL-33 in endothelium during allergic airway inflammation

Maile K. Hollinger, Chanie L. Howard, Donna C. Decker, Kelly M. Blaine, Ivy Aneas, Emily M. Grayson, Tania E. Velez, Fernando A. Oliveira, Riley T. Hannan, Daniel F. Camacho, Phil A. Verhoef, Cara L. Hrusch, Rebecca S. Griffes, Jeffrey M. Sturek, Marcelo A. Nobrega, Nathan Schoettler, Anne I. Sperling

Supplemental Figures

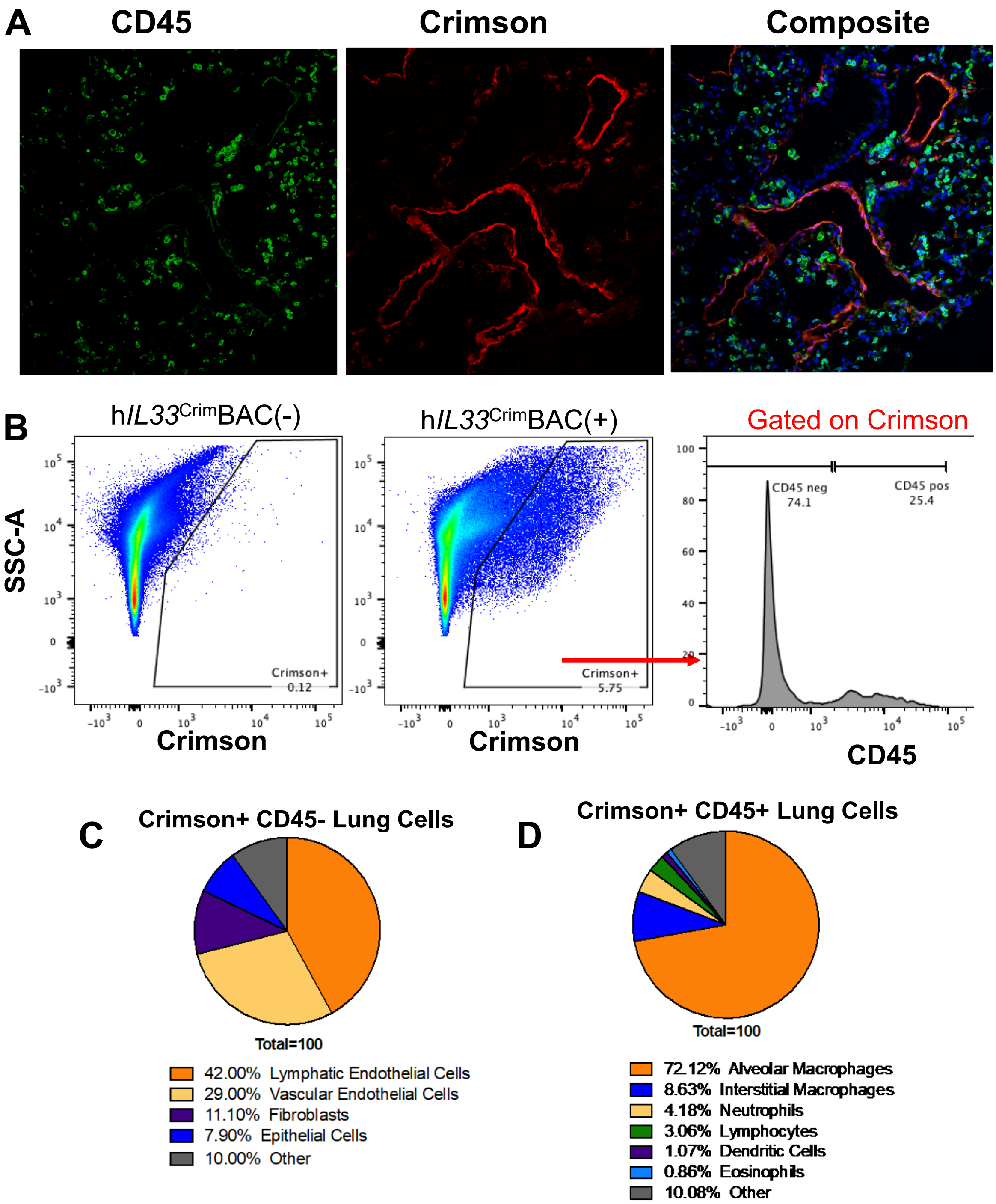

Supplemental Figure 1: *hIL33* reporter expression is primarily restricted to CD45(-) lung cells. (A) Confocal microscopy of lung sections from a 6–8-week-old h*IL33*^{Crim}BAC mouse. Green: CD45; red: Crimson (hIL33 reporter); blue: Hoescht nuclei staining. (B) Representative flow cytometry plots of cells from whole lung digests of a h*IL33*^{Crim}BAC(+) (right) and h*IL33*^{Crim}BAC(-) littermate control (left). Crimson(+) cells were gated on CD45(+) and CD45(-) for subsequent analysis (far right). (C & D)) Breakdown of Crimson expression in CD45(-) and CD45(+) lung cells from h*IL33*^{Crim}BAC(+) mice.

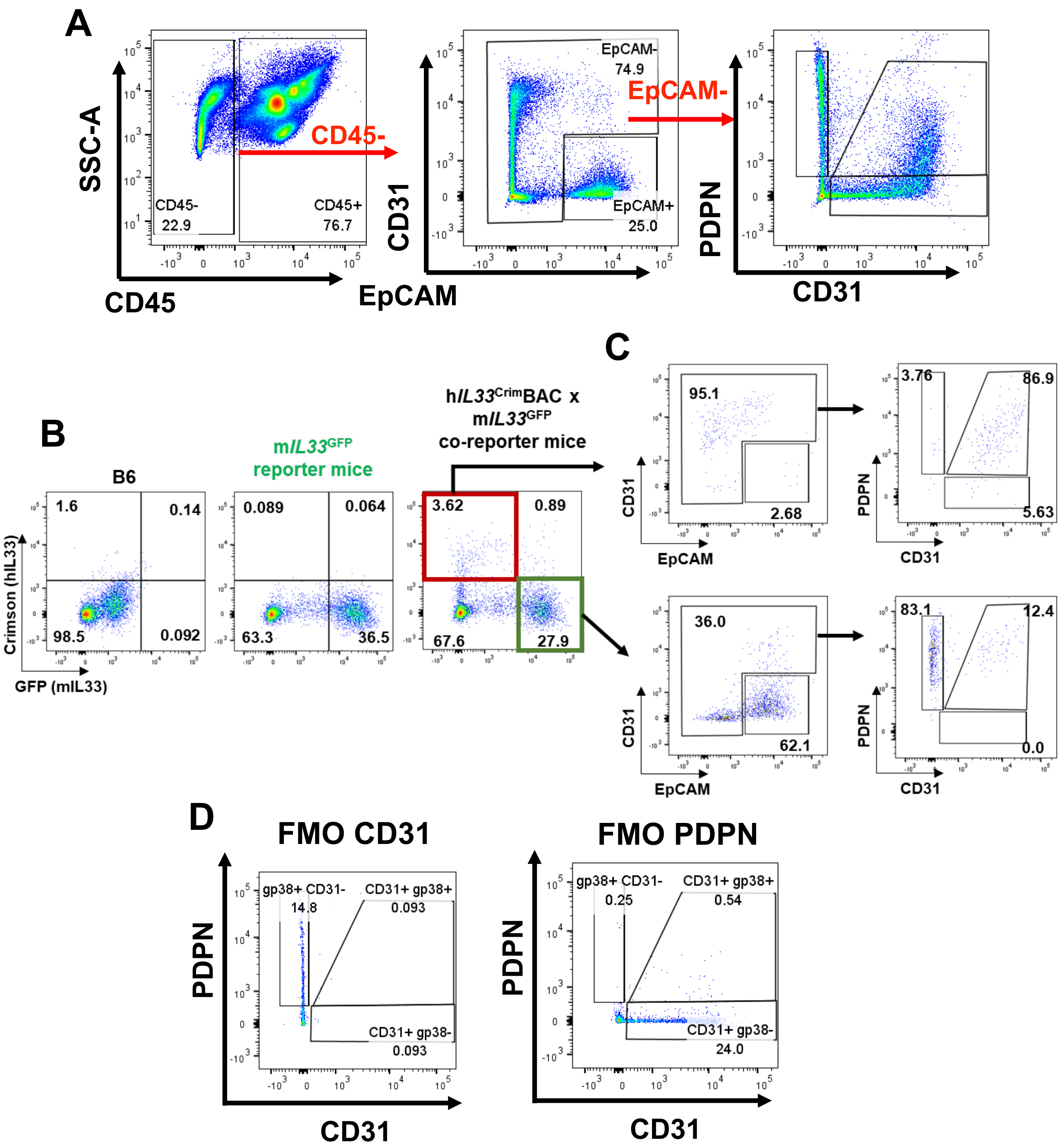

Supplemental Figure 2: Gating strategy to identify Crimson-expressing CD45(-) lung stromal cell subsets. (A) Live, CD45(-) cells (left) were gated to identify lung epithelial, endothelial, and fibroblast populations. EpCAM(+), CD31(low) cells were classified as epithelial cells (middle). All remaining cells were then divided into CD31(+)PDPN(-) vascular endothelial cells (VECs), CD31(+)PDPN(+) lymphatic endothelial cells (LECs), and CD31(-)PDPN(+) fibroblasts (right). (B) Crimson expression in LECs from a C57BL/6 mouse (left), Crimson-negative h*IL33*^{Crim}BAC x m*IL33*^{GFP} mouse, and Crimson-positive h*IL33*^{Crim}BAC x m*IL33*^{GFP} littermate (right). Red box indicates Crimson single-positive, CD45(-) cells; green box indicates GFP-positive, CD45(-) cells. (C) Breakdown of Crimson expression (top) and m*IL33*^{GFP} expression (bottom) from a Crimson-positive h*IL33*^{Crim}BAC x m*IL33*^{GFP} mouse using the gating strategy defined in (A). (D) Fluorescence minus one (FMO) controls for CD31 and PDPN staining.

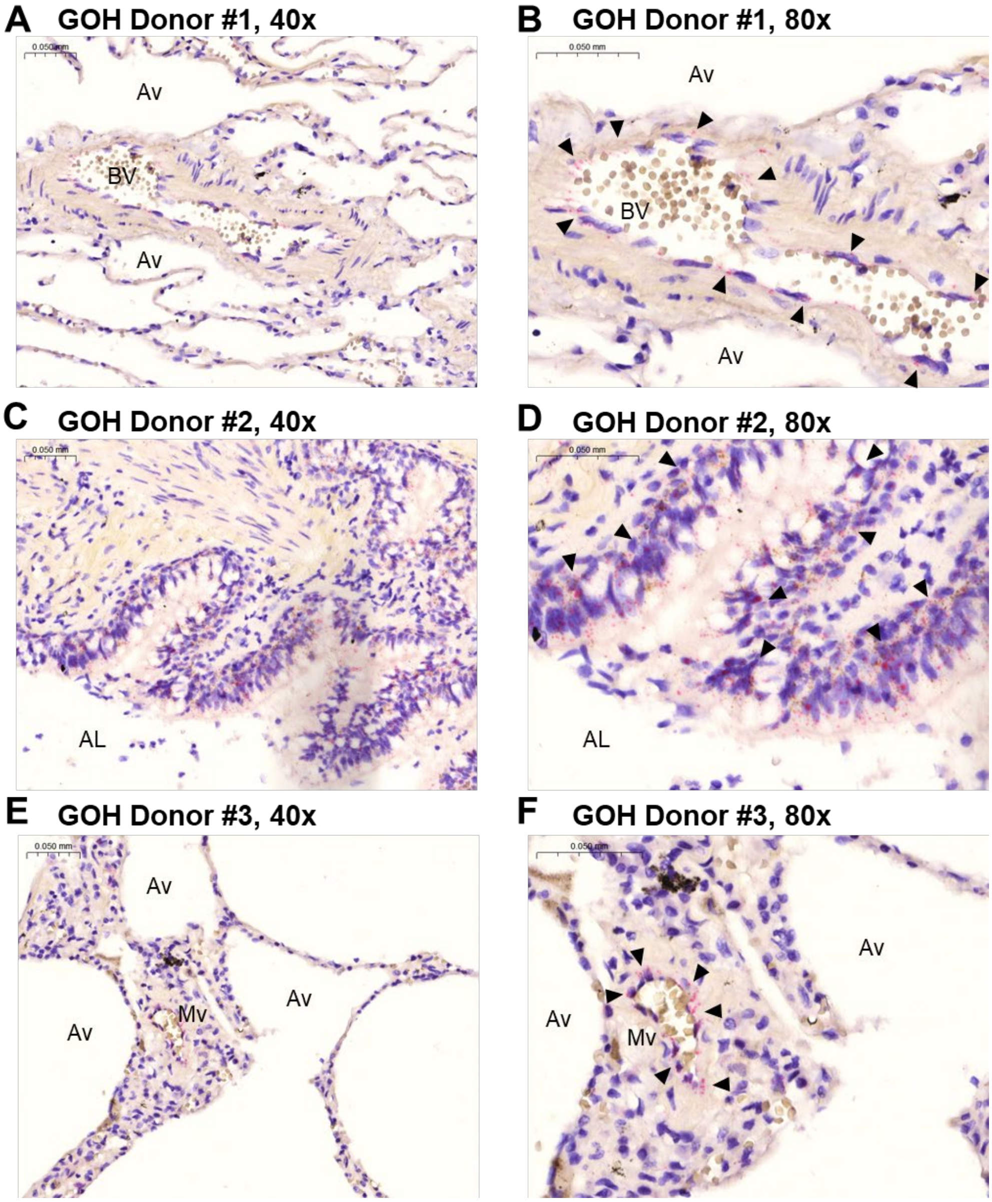

Supplemental Figure 3: RNAscope staining for *IL33* transcript in human lungs from Gift of Hope organ donors. (A) 40x field showing a blood vessel (BV) surrounded by alveolar spaces (Av). (B) 80x field of blood vessel from (A) with anti-*IL33* probes (pink) in the endothelium lining the vessel (black arrows). (C) 40x field of a bronchiole with basal epithelium. Airway lumen is indicated by "AL". (D) 80x field of bronchiole from (C) with anti-*IL33* probe along the basal epithelium (black arrows). (E) 40x field of a microvessel (Mv) surrounded by alveolar spaces (Av). (F) 80x field of microvessel from (E) with anti-*IL33* probe in endothelial cells lining the vessel.

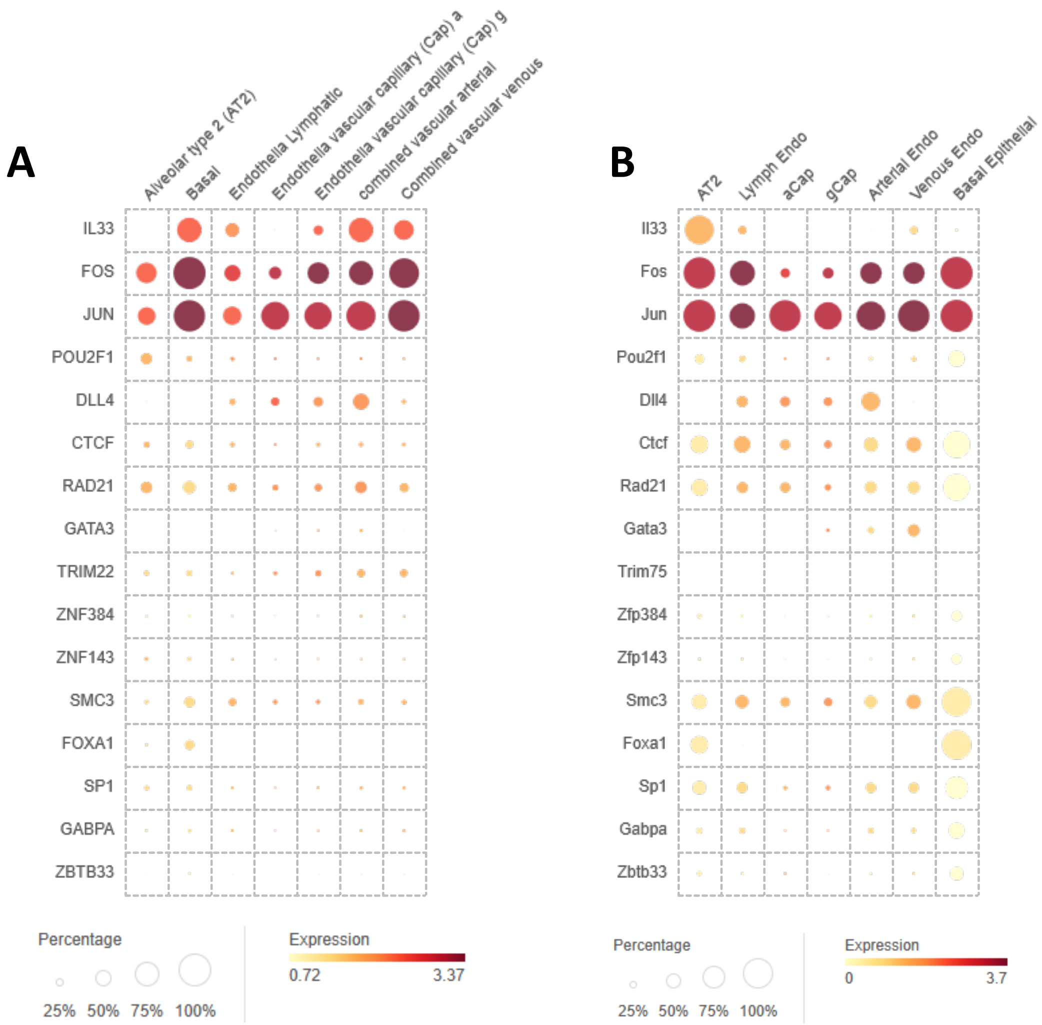

Supplemental Figure 4: Comparison of transcription factor expression across cell compartments in humans and mice. (A) Gene dot plot for human transcript levels of *IL33* and transcription factors with demonstrated binding to the 5 kb region upstream of the human *IL33* gene by ENCODE. Plot was generated using data (n=10) from Madisson et al. *Nat Genet* 2022 (PMID: PMC9839452). (B) Gene dot plot for murine transcript levels of *Il33* and orthologues to the transcription factors in (A). Plot was generated using data from Curras-Alonso et al. *Nat Comm* 2023 (PMID: PMC10147670) (n=5 control mice). AT2, alveolar type 2 epithelium; Lymph Endo, lymphatic endothelium; aCap, capillary (arterial); gCap, capillary (venous); Arterial Endo, arterial endothelium; Venous Endo, venous endothelium.

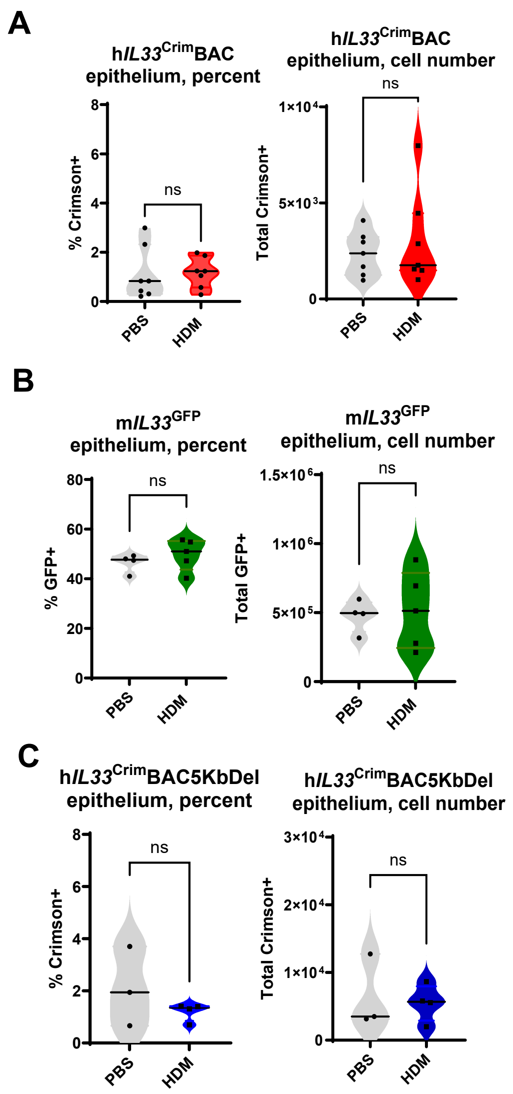

Supplemental Figure 5: Human and murine IL33 reporter expression in lung epithelium is unaffected by HDM treatment. (A) Crimson expression in lung EpCAM(+) epithelial cells from h*IL33*^{Crim}BAC mice treated with HDM as in Figure 3, as percentage of total Crimson(+) epithelial cells (left) and total number of Crimson(+) epithelial cells (right). (B) IL33-GFP expression in epithelial cells from the lungs of m*IL33*^{GFP} mice treated as in Figure 3. (C) Crimson expression in epithelial cells from the lungs of h*IL33*^{Crim}BAC5KbDel mice treated as in Figure 3. ns, not significant by unpaired t test.
